# Goal Setting for Healthy Brain Aging in Older Adults: What Matters Most to Participants of the AgeWISE-AP Program

**DOI:** 10.1093/geroni/igaf122.3779

**Published:** 2025-12-31

**Authors:** Jaye McLaren, Andrew Nguyen, Emily Metcalf, Maureen O’Connor

**Affiliations:** VA Bedford Healthcare System, Bedford, Massachusetts, United States; Department of Veterans Affairs, Bedford, Massachusetts, United States; VA Bedford Health Care System, Bedford, Massachusetts, United States; Bedford Veterans Health Care System, Bedford, Massachusetts, United States

## Abstract

Reducing cognitive decline is described as an important aspect of successful aging. VA supported Age Friendly Healthcare Systems focus on the 4Ms framework (What Matters Most, Medication, Mentation, and Mobility) to improve quality of care and care delivery for older Veterans. The Aging Well through Interaction and Scientific Education– Action Plan program (AgeWISE-AP) emphasizes the What Matters Most element of the 4Ms as we collaborate 1-1 with Veterans to identify individualized lifestyle goals that support positive brain health outcomes. AgeWISE-AP is comprised of two components: an initial 12-week group to educate participants on brain aging and health factors followed by 8 individual sessions supporting participants in the creation and implementation of client-centered goals within the domains of healthy brain aging (e.g. diet, exercise, personal development, socialization etc.) outlined in the Whole Health Circle of Brain Health. Led by a Brain Health Interventionist (BHI), AgeWISE-AP bridges the gap between education and action. Here we report on 36 participants, who completed a total of 282 individual sessions, making 120 lifestyle goals within the 8 domains of healthy brain aging. Using case examples, we will illustrate emerging trends regarding domain selection and goal creation for participants to date. Understanding broadly what matters most to Veterans (their “why”) as well as, more specifically, what personal interests, activities, or values drive them to consistently participate in lifestyle behavior changes for healthy brain aging (their “what”) is essential to the success of this intervention.

